# A qualitative study of the perceived effects of blue lights in washrooms on people who use injection drugs

**DOI:** 10.1186/1477-7517-10-22

**Published:** 2013-10-08

**Authors:** Alexis Crabtree, Gareth Mercer, Robert Horan, Shannon Grant, Tracy Tan, Jane A Buxton

**Affiliations:** 1 University of British Columbia, Vancouver, Canada; 2 British Columbia Centre for Disease Control, Vancouver, Canada; 3 University of Saskatchewan, Saskatoon, Canada; 4 University of Calgary, Calgary, Canada; 5 Memorial University of Newfoundland, St. John's, Canada

**Keywords:** Harm reduction, Injection drug use, Public injection, Fluorescent blue lights, Internalized oppression

## Abstract

**Background:**

Blue lights are sometimes placed in public washrooms to discourage injection drug use. Their effectiveness has been questioned and concerns raised that they are harmful but formal research on the issue is limited to a single study. We gathered perceptions of people who use injection drugs on the effects of blue lights with the aim of informing harm reduction practice.

**Methods:**

We interviewed 18 people in two Canadian cities who currently or previously used injection drugs to better understand their perceptions of the rationale for and consequences of blue lights in public washrooms.

**Results:**

Participants described a preference for private places to use injection drugs, but explained that the need for an immediate solution would often override other considerations. While public washrooms were in many cases not preferred, their accessibility and relative privacy appear to make them reasonable compromises in situations involving urgent injecting. Participants understood the aim of blue lights to be to deter drug use. The majority had attempted to inject in a blue-lit washroom. While there was general agreement that blue lights do make injecting more difficult, a small number of participants were entirely undeterred by them, and half would use a blue-lit washroom if they needed somewhere to inject urgently. Participants perceived that, by making veins less visible, blue lights make injecting more dangerous. By dispersing public injection drug use to places where it is more visible, they also make it more stigmatizing. Despite recognizing these harms, more than half of the participants were not opposed to the continued use of blue lights.

**Conclusions:**

Blue lights are unlikely to deter injection drugs use in public washrooms, and may increase drug use-related harms. Despite recognizing these negative effects, people who use injection drugs may be reluctant to advocate against their use. We attempt to reconcile this apparent contradiction by interpreting blue lights as a form of symbolic violence and suggest a parallel with other emancipatory movements for inspiration in advocating against this and other oppressive interventions.

## Background

The use of injection drugs in public spaces is associated with individual (physical, psychological) and societal harms. Physical harms, such as abscess and damage to veins, become more likely in settings where injecting is rushed and hygiene is compromised [1-8]. Lack of privacy compounds the shame that many people who use drugs feel [9,10] and communities where public drug use occurs become marked and subject to stigma [11-13]. Strategies to reduce public injection drug use, or to reduce the harms associated with injecting in public, could benefit people who use injection drugs and the communities where they live.

Attempts to reduce harms associated with public injecting include strategies that support people who use injection drugs (supervised injection sites, provision of housing) and dispersal strategies (intensified policing, changes to the built environment). In this article we focus on a single dispersal strategy, the installation of blue lights in public washrooms. Here we define the term “public washroom” as including washrooms located in not-for-profits, community facilities, and privately owned businesses, but not in private homes. Blue lights emit an intense blue-coloured light, with the purpose of visually obscuring superficial veins [14]. A qualitative study from the United Kingdom of people who use injection drugs' experiences with blue lights suggested that, while blue lights do make it more difficult for them to find their veins, for some this would not deter attempted injection. Potential reasons for this lack of deterrent effect included scarcity of alternative injection locations and confidence in their injecting ability [15]. Theoretically, blue lights could compound the risk of injecting in public washrooms by increasing the probability that people who use injection drugs will miss the target vein and inject into surrounding tissue and by promoting other unsafe practices such as deep vein injecting (which is done without visual identification of the vein and therefore is not deterred by blue lights) [16]. Aside from the United Kingdom study, there is no formal research into the harms of installing blue lights in public washrooms. The existing research has indeterminate applicability to the Canadian setting, since the context of injection drug use differs between the United Kingdom and Canada. In particular, in one of our study sites, the existence of a supervised injection facility (SIF) provides an alternative to injecting in washrooms, and the existence of blue lights in nearby washrooms may influence people who use drugs' decision-making about visiting the SIF.

The purpose of this study was to engage people who are current and former users of injection drugs from two cities in British Columbia, Canada, to share their perceptions of the potential harms and benefits associated with blue lights in public washrooms. We aim for our results to inform harm reduction practice regarding blue lights.

## Methods

Between January and March, 2011, a sample of people identifying as current and former users of injection drugs was recruited purposively through drug user community advocacy groups in Vancouver and Abbotsford, British Columbia (the Vancouver Area Network of Drug Users (VANDU) and the BC Yukon Association of Drug War Survivors, Abbotsford Chapter). Board members of these organizations identified individuals who had experience injecting drugs in public places and invited them to speak privately with the researchers to learn more about the study. This recruitment method was chosen over the use of intensive screening questions or observation of track marks in order to create a respectful research environment in which people feel they are participants rather than subjects, and to take advantage of the expertise of the collaborating advocacy organizations. Participants were required to be aware of the issue of blue lights in public washrooms, though they were not required to have personally experienced injecting in a bathroom fitted with a blue light. This allowed us to explore reasons why a person who encounters blue lights might chose to inject elsewhere, in addition to exploring perceptions and decision-making among those who have injected under blue light conditions. We had not planned to explore how people who use injection drugs view themselves in relation to people who do not use drugs, but this was a persistent theme identified in our interviews and we explore it in this article to the extent allowed by our data.

Participants’ perceptions of the impacts of blue lights in washrooms were gathered using approximately half-hour long, semi-structured interviews conducted by the first five authors. Interviews were conducted in private rooms made available by the two community advocacy groups. At the beginning of each interview, written informed consent was obtained and participants were offered contact information for the study principal investigator and for a counselor who would be available to speak with them following their interview. Participants were offered a nominal honorarium to thank them for their involvement in the study. All names presented are pseudonyms.

Interviews were recorded and transcribed verbatim, then organised using qualitative analysis software (NVivo 9). The analytic approach of interpretive description was followed [17], whereby vocalized thoughts were coded into a set of concept categories. Concept categories were further clustered into an overarching set of themes. These themes were presented to representatives of the drug user advocacy groups in Vancouver and Abbotsford for member checking and their comments incorporated into the final analysis. This method was chosen with the aim that the “interpretive description” derived from the final thematic structure could be used to inform harm reduction practice and policy.

Initial transcripts were coded together by all five interviewers so that consensus could be reached on the assignment of thoughts to concept categories and the structure of these categories within the overarching themes. Later interviews were coded by each individual interviewer using the existing thematic structure, adding new concept categories and themes where they emerged. Interviews were conducted until there was general agreement amongst all interviewers that data saturation had been achieved, defined as the point where no new themes emerged from the most recent round of interviews.

Approval for this study was obtained from the Behavioural Research Ethics Board of the University of British Columbia.

## Results

### Characteristics of the participants

Eighteen participant interviews were conducted. Eight interviews were conducted in Abbotsford and 10 in Vancouver. Eight participants reported current injection drug use, while ten reported no longer using injection drugs. Six participants were women and 12 were men; the sex distribution was similar among participants from the two cities. All participants knew about the practice of blue lights being installed in public bathrooms, and 16 described situations in which they had personally attempted to inject in a blue-lit bathroom.

### Preferred geographic locations for using injection drugs

To contextualize the participants’ perceptions of the impacts of blue lights, we first sought to explore the criteria that made particular geographic locations preferred places for them to inject drugs. The majority of participants described cleanliness and being indoors as important factors. Two participants specifically mentioned preferring locations with “proper lighting.” Two key themes to emerge from our exploration of preferred injection location were “privacy” and “immediacy.”

#### Privacy

A majority of participants told us that finding a location that afforded privacy was a priority. Reasons included wanting to avoid interruption and wishing not to be judged by others or “labeled as a junky” [Daniel, Vancouver]. The desire for privacy was strongly tied to a sense of shame about being observed while using illicit drugs. Bruce (Abbotsford) in response to a question about being seen while injecting, answered, “I don’t know, I feel kind of bad for them, actually, you know, like, I shouldn’t be there shooting dope, like, you know, it’s just-- it’s not right, I don’t think.” Bruce's response includes a moral judgment ("it's not right") as the basis for his discomfort with being observed injecting, and includes an implication that non-injectors should not have to witness his actions. Several participants mentioned being seen by children as a particular concern, reflecting an assumption that simply witnessing drug use would cause children harm:

*“I*’*m invading their space and I*’*m doing something that*’*s not sociably acceptable*. *And I almost feel bad about it*, *felt bad about it*, *you know*, *a little bit*. *Had a child or something like that walked in and*-- *I impacted their life somehow by them experiencing seeing me doing it or the after effects of me doing it*, *which has never happened*, *but if it did I would feel worse about it than I do*.” [*Greg*, *Vancouver*]

For most, a desire for privacy was expressed as a preference for locations where they could be alone. Peter explained that having someone else there “kind of destroys the feeling of getting high,” while Sally told us: “It was a personal thing to me. I didn’t want that image in anybody’s head of me… sitting there injecting.” However, more than half of the interviewees also included locations where there are other people around in their discussion of private places. Places mentioned included drop-in centers, like VANDU, and the supervised injection facility in Vancouver, Insite. The sense of privacy at these places was linked to feeling less exposed to judgement when using injection drugs. For example, describing VANDU, Natalie (Vancouver) explained, “When I’m coming here I don’t feel like I’m being treated like just another junky. I feel like I’m just being treated like I’m just another human being.” Only three participants told us that they specifically preferred injecting in the company of other people, but it is notable that one participant mentioned needing to be around other people when he injects because he sometimes requires assistance. Vancouver's SIF does not accommodate assisted injecting, so needing assistance to inject further limits options for places to consume injection drugs [18].

#### Immediacy

Although the vast majority of participants had definite criteria for preferred injection locations, most (15/18) made it clear that the locations where they use injection drugs are often suboptimal and selected under pressure. This is particularly the case when they are experiencing withdrawal symptoms (“dope-sick”):

*“I mean*, *whatever*-- *if I*’*m dope*-*sick then it really doesn*'*t matter where I do it*. *I mean*, *I would do it in front of a cop car*, *fix myself*.” [*Natalie*, *Vancouver*]

“*Oh*, *when I*’*m heavy in my addiction I don*’*t care*, *right*. *It*’*s just* -- *I try to get it in me*. *I don*’*t care*. *I*’*ve done it right on Main Street and Hastings* [*a major intersection in Vancouver*], *actually*. *Even done it out here* [*in Abbotsford*] *on the street*, *right*.” [*Bob*, *Abbotsford*]

*“I never do decide* [*where to inject*]. *Just wherever*, *right*. *If I score here*, *and*, *you know*, *the obsession is there*…*I always go around the corner from where I scored and use*.” [*Bob*, *Abbotsford*]

In our analysis of participants’ perceptions of the impacts of installing blue lights in public washrooms, we found it critical to keep in mind the apparent conflict between the ideal of accessing private, non-judgmental places to use injection drugs and the frequent reality of accessing the closest, easiest location. Participants suggested that in circumstances when they feel they urgently need to inject, they would be more likely to use public spaces like bus stops, alleys, and public washrooms.

#### Washrooms as a compromise between privacy and urgency

Finally, in considering preferred locations for using injection drugs, we sought to specifically understand participants’ beliefs about using public washrooms. All of the participants had accessed public washrooms as locations for using injection drugs, but about half specifically said that these were not preferred places for them. Interestingly, two participants described public washrooms as having the benefit of being relatively private:

"…*it*'*s private*, *you know what I mean*. *There*'*s one place where you go*, *just where you go in there*, *no one else can*. *You know what I mean*? *You*'*re okay in there* '*till you come out*, *right*.” [*Daniel*, *Vancouver*]

“*No*, *I don*'*t see any harm in using drugs in the bathroom* '*cause you*'*re only there and then you*'*re gone*, *right*. *It*'*s better than letting people use them out in the alley*, *out in front of kids*….” [*Pierre*, *Vancouver*]

Conversely, three participants described public washrooms as feeling “unsafe” because of a lack of privacy. As an example, Sam from Abbotsford told us: "I feel trapped in a public washroom. You come out and you're all wasted and there's people standing around...."

The majority of participants described public washrooms as locations they would use for their accessibility (“it’s easy and handy” [Sam, Abbotsford]), but usually only when they need somewhere to inject urgently (“…if I was out and I was sick, I’d go to a public washroom.” [Marg, Abbotsford]). Based on the perceptions of a smaller number of the participants, public washrooms may also be important locations for people who do not have a private space of their own, especially people who are homeless.

"*If I was homeless*, *which happened from time to time*, *I would go to*, *like*, *the church bathroom or the Carnegie* [*library*] *bathrooms a lot*." [*Greg*, *Vancouver*]

Understanding public washrooms as locations for urgent injecting and as places used by people with few private alternatives helped us to interpret participants’ responses to blue lights.

### The perceived effectiveness of blue lights as a deterrent to injecting in public washrooms

One aim of this study was to explore what people who use injection drugs understand to be the purpose of installing blue lights in public washrooms, and whether they think that purpose is being effectively achieved.

Except for one of the participants who had not attempted to inject in a blue-lit bathroom, there was unanimous recognition that the aim of installing blue lights is to make it difficult to do injections, thereby to “deter people from using [intravenous] drugs in [the] bathroom.” [Marg, Abbotsford] About half (7/18) of the participants explicitly described the aim of blue lights as being to make it “so you can’t see your veins.” [Daniel, Vancouver] The remainder gave more general descriptions such as, “…it makes everything hard to see and disorienting,” [Greg, Vancouver] “everything looks the same colour,” [Steve, Vancouver] and they make it “…hard to make the injection work.” [Bob, Abbotsford]

In interpreting the discussions about the effectiveness of blue lights we observed an important distinction: their effectiveness at making it difficult to inject versus their effectiveness as a deterrent. All those who had tried agreed that it is more difficult to inject under blue lights. The discussion of their deterrent effect was much more complex, often requiring consideration of the situation in which injection drug use is taking place. A majority (15/18) of participants said that they would generally try to avoid public bathrooms where they knew blue lights had been installed. At the opposite extreme, three participants made comments suggesting that they were entirely undeterred by blue lights. For example:

"*It didn*’*t really make a difference for me*. *It*’*s almost like a challenge*." [*Alice*, *Abbotsford*]

“*It doesn*’*t seem to bother me because I always know where I*’*m going*. …*You get used to it after awhile*.” [*Peter*, *Abbotsford*]

Even among those who said they would try to avoid blue-lit bathrooms, almost half (6/15) described strategies they would use to overcome some of the inconvenience imposed by blue lights. These strategies include: preparing drugs and loading syringes before entering the bathroom; bringing other light-sources like candles, lighters, or flashlights with them into the bathroom; injecting by “feel”; relying on the “flash” of blood entering the needle to know when they were in a vein; injecting into a muscle rather than a vein; or even “step[ing] out into the hallway and do[ing] it there.” [Richard, Abbotsford] Importantly, women and veteran injectors, who were described as having smaller or more difficult to access veins, were identified by participants as having particular difficulty adapting their practices to successfully inject under blue lights.

A key finding was that half (7/15) of those who said they generally avoid bathrooms with blue lights explained that they would not be deterred from injecting in these bathrooms if they perceived that they had no alternative.

“*When push comes to shove*, *it would mean no difference to me*, *really*. *If I needed the bathroom and it was there and it was*, *like*, *my only choice in the area*, *I wouldn*’*t think twice about going there again*. *I*’*m sure I could do what I needed to do in there again*.” [*Greg*, *Vancouver*]

“*If I could find something better*, *yes* [*I would avoid blue lights*]; *if it was my last resort*, *I*’*d try*. *Simple*.” [*Marg*, *Abbotsford*]

In interpreting these assertions it is useful to remember that most of these statements came from participants who described a preference for avoiding public bathrooms as injection locations, but acknowledged that they offer a reasonable compromise between privacy and accessibility in situations when they need somewhere to inject immediately, especially when they are experiencing withdrawal symptoms. Taken together these findings suggest that blue lights, while effective at making injecting more difficult, would not deter most of our participants from injecting if the need were urgent -- in other words, blue lights would not deter injecting in precisely those moments when injectors are most likely to balance privacy and immediacy by using a washroom for injection in the first place.

### The perceived negative consequences of installing blue lights

The next step in our exploration of the perceived effects of installing blue lights in public washrooms was to ask participants whether they felt there to be any negative consequences to the practice. The responses can generally be organized as relating to either the direct effects of making injecting more difficult or to the effects of dispersing injection drug use to other public places.

About half of the participants believed that, by making it harder to see veins, blue lights could make injecting more dangerous. A number suggested different ways that a person struggling to find a vein while injecting under blue light could be more likely to damage her/his body, including causing more needle scars, damaging an artery or nerve, starting an abscess or over-dosing. In particular, many felt that a person who is desperate enough to inject in a bathroom with a blue light is not going to give up easily, and is thus especially vulnerable to harming her/himself. These findings suggest that blue lights may hinder people’s ability to observe the safer injection practices promoted by harm reduction practitioners. One participant also suggested that blue lights could make a person more likely to spill blood or leave her/his used needle in the washroom. Although a minority opinion, we feel it is justified to raise it because a number of other participants felt that installing needle-disposal bins in public washrooms, (as opposed to focusing on deterring use in that location with interventions such as blue lights) would be a more effective means of reducing drug-use-related litter.

About one third of participants spoke about the dispersal effect of blue lights. These individuals tended to respond to the question of whether blue lights deter injecting with variations on the idea that, if blue lights “inhibit people from successfully I.V. drug using in the [washroom]-- they just go somewhere else where they can [inject].” [Daniel, Vancouver] These participants appeared to share the perception that washrooms offer safer injection locations than the alternatives for people who are in the situation of needing to access a public place for injecting. The following quotes clearly demonstrate this concern:

“*The alley*. *That*’*s the thing*, *you know*, *doing that*, *you send them out to unsafe*-- *really unsafe places*.” [*Sally*, *Vancouver*]

“…*outside*-- *it*’*s just sometimes*, *it*’*s not a very clean thing to do outside*-- *water wise*, *you know*. *I*’*ve seen people suck up mud puddle water and stuff like that*.” [*Ben*, *Abbotsford*]

In addition to making injection drug use more difficult and potentially harmful than in public washrooms, these locations offer less privacy. Blue lights may, therefore, be expected to make public injection drug use a more visible, shameful experience.

Almost all Abbotsford participants mentioned their desire for a local supervised injection facility and discussed the role it could play in providing a safer alternative to public washrooms (especially those that are blue-lit). For example, when asked what could be done in addition to blue lights to reduce drug use in public washrooms Emma [Abbotsford] told us: “Well, a safe injection site, definitely. But Abbotsford, I mean…it’s a million miles away from, you know, for that to happen.” None mentioned potential problems or drawbacks to such a facility. In contrast, Vancouver participants suggested the supervised injection facility did not usually provide an adequate alternative to the dispersal effects of blue-lit washrooms. Although generally valuing the facility, they suggested several barriers to its use: distance or the need for more than one facility in several areas of Vancouver; wait times to use the facility (potential clients are asked to wait in queue if the facility is at capacity); rules against assisted injecting; and lack of privacy. Regarding the experience of injecting at the supervised injection facility, Steve [Vancouver] commented, "A private bathroom is the best 'cause at Insite, even, I feel perfectly safe but I always feel like someone’s watching me. Which they have to, right." Overall, Vancouver-based participants' statements suggest that the supervised injection facility does not consistently meet the need for immediacy and privacy that encourages individuals to use public washrooms for injecting.

These findings, taken together with those presented in the previous section, suggest that installing blue lights in a public washroom is unlikely to have the desired effect of preventing all drug use there, but it may preclude the use of that washroom as a safe place for injecting by those who need it most.

### Participant recommendations in favour of blue lights despite their negative effects

Although a number of negative effects of blue lights in washrooms were identified by participants, most (13/18) indicated they were not opposed to their use or even recommended them. Reasons given included supporting business owners' rights to use their property as they see fit and purportedly making washrooms safer for patrons:

*“I don*’*t believe when a person goes out to a public place that they should have to be worried*-- *if a person*’*s going to be that blatantly open about*, *like*, *about using drugs in public*, *then they*’*re sometimes not going to be worried about leaving their paraphernalia behind*. *And they*’*re going to endanger other people as far as I*’*m concerned*.” [*Emma*, *Abbotsford*]

Implicit in this statement is that once a person chooses to use an establishment’s washroom for the purposes of injecting drugs, they cease being a “patron" and thus give up the privilege of using the washroom. Kevin (Vancouver) told us, “it’s not for that purpose. It’s for patrons. It’s for, you know, people in the restaurant or in the gas station. It’s not for injecting.”

Almost half (7/18) of participants both identified harms from blue lights and made positive statements about their use. This is an important finding, as shows a devaluing of people who use injection drugs' health in favour of the experience of non-drug users.

## Discussion

The use of blue lights in public washrooms has been interpreted by others as a form of symbolic violence against people who use injection drugs [15]. Symbolic violence describes how power is used to control and discipline people in ways that seem natural to both sufferers and perpetrators [19]. Our results support this interpretation and highlight how people who use injection drugs may come to accept and even support such practices through a process of internalized oppression.

Symbolic violence reinforces existing axes of oppression and serves a political purpose. When attention is directed to drug use as criminal, shameful, and a potential vector of disease, it is directed away from the harms of the drug war and from those who benefit economically and politically from its continuation [20]. As well, by making exclusion seem natural or for the benefit of people who use drugs, actions of symbolic violence can cause them not to resist their own oppression and even to perpetuate it.

In keeping with the practical and incremental goals of harm reduction, many practitioners would laud people who use drugs for choosing a location to inject that is potentially quiet, unrushed, well-lit, and equipped with running water (while, of course, working towards the day when injecting drugs in public washrooms is no longer the best of a constrained set of choices). In our research, participants advocated for the use of blue lights despite identifying harms from this practice. Their support of blue lights was based not on fear of speaking out against them, but of a deep-seated sense of shame in their use of public washrooms to inject. We therefore identify this as an example of internalized oppression in keeping with the symbolic violence of blue lit washrooms.

This research has several implications for harm reduction practitioners. First, participants identified significant harms from blue lights in public washrooms, including physical damage to veins and surrounding tissue and displacement to riskier spaces for injection. The latter may increase the psychological harms of public injection drug use by making it more visible, enhancing the accompanying sense of shame. Those working in harm reduction should be aware of the negative impacts of blue lights and be prepared to advocate against their use.

Second, confirming other qualitative and quantitative work [21-24], we found that supervised injection facilities do not always meet the needs of people who use injection drugs for privacy and immediacy. This means that the deterrent effect of blue lights will not necessarily result in injecting in safer locations, even when supervised injection facilities are available. Therefore, while the expansion of supervised injection facilities is desirable to reduce other drug-related harms (and was called for strongly by our participants in the community that lacked a SIF), the presence of SIFs should not be seen as eliminating the harms imposed by blue-lit washrooms.

Finally, our results indicate that internalized oppression may inhibit people who use injection drugs from acting to challenge practices that cause them harm, such as installing blue lights in washrooms. Self-advocacy by people who use drugs has been an important force in the harm reduction movement [25-31], but has been limited by lack of funding, legal concerns, and the prioritization of survival needs by people who use drugs [32-36]. We suggest that internalized oppression adds to these difficulties by discouraging people who use drugs from engaging in self-advocacy and even leading them to defend the use of harmful practices. Other liberatory struggles (for example, the women's movement) have involved a process of consciousness-raising to challenge internalized oppression [37,38]; those working with people who use drugs may benefit from exploring how similar techniques could be incorporated into their practices.

Several limitations to this research should be noted. The recruitment strategy selected for participants who were connected to other people who use drugs and to drug users' advocacy organizations. People who use drugs in relative isolation (i.e. without others having knowledge of their injection drug use) possibly have more complex needs regarding privacy and choices of injecting location. In addition, we recognize those without access to private places to inject (particularly homeless people) may experience blue lights and their effects differently; few participants in our sample were homeless, and further research with this population may yield additional insights.

In summary, the installation of blue lights in public washrooms is an example of a structural intervention aimed at reducing public injection drug use, which, superficially, may appear less oppressive than many other drug-use dispersal strategies. Our analysis of the perceptions of people who use injection drugs supports previous findings that installing blue lights is unlikely to deter injection drugs use in public washrooms, and may increase drug use-related harms. Importantly, we support the suggestion that blue lights are a form of symbolic violence: they aim to tacitly control people who use injection drugs, forcing them to make choices about places to inject that are not in their best interests, and imposing upon them the belief that their own well-being is secondary to the interests of those responsible for installing the blue lights. We encourage harm reduction practitioners to consider the potential harms of blue lights and to advocate against their use. Practitioners working on advocacy projects with people who use injection drugs may find it valuable to facilitate opportunities to promote their clients' sense of self-worth and to illustrate the ways in which harmful drug-use dispersal interventions are forms of oppression.

## Competing interests

The authors declare that they have no competing interests.

## Authors’ contributions

AC and JB conceived of and designed the study. AC, GM, RH, SG, and TT collected and analysed data and wrote sections of the manuscript. JB oversaw data collection and analysis and revised the manuscript. All authors read and approved the final manuscript.
